# Supplementary material for: Spreading to localized targets in complex networks
Source: Sci Rep. 2016 Dec 14;6:38865. doi: 10.1038/srep38865 (PMC5155210; doi:10.1038/srep38865)
Supplement: Supplementary Information [file srep38865-s1.pdf]

Supplementary Information

Spreading to localized targets in complex networks

Ye Sun, Long Ma, An Zeng and Wen-Xu Wang

Supplementary Note 1: The comparison between a linear model and RLP method.

We propose a simple linear model considering nodes' degree and average distance to targets. Mathematically, the formula for the linear model reads

$$S_i = \theta \frac{k_i}{k_{max}} + (1-\theta)\frac{\langle d\rangle_{max}}{\langle d_i\rangle} \tag{1}$$

where θ is a parameter between 0 and 1. k_i is the degree of node i. k_{max} is the largest degree of all nodes. $\langle d_i\rangle$ is the average shortest distance from node i to all targets. $\langle d\rangle_{max}$ is the largest $\langle d_i\rangle$ of all nodes.

We compared the rank correlation coefficient τ based on this method with the results of the RLP method in Fig. S1. Two networks are considered: WS and BA. In each network, we select 50 target nodes. In Fig. S1ab, the targets are randomly located in the networks. In Fig. S1cd, the targets are randomly located within distance L=2 to a center node. The red rhombus line represents the results of RLP method and the blue circle line represents the results of the linear model. One can clearly see that the linear combination of degree and average distance can indeed result in a higher coefficient τ. However, the results of RLP method are better than that of this linear model under different values of θ.

Supplementary Note 2: The comparison between the RLP method and the centrality methods when all nodes are target nodes.

We compare the accuracy τ between the RLP method and the centrality methods (degree, betweenness and k-core) under different infection rate λ in Fig. S2. We consider the case where all nodes are targets in four different networks. In WS and BA networks (Fig. S2ab), we do not show the results of the k-core method as the k-shell values of all the nodes in these two networks are almost the same. The results in each figure is obtained by averaging over 5000 independent realizations. In Fig. S2, one can see that the RLP method

outperforms other centrality methods, especially when the infection probability is near the critical infection probability λ_c in these artificial and real networks.

Supplementary Note 3: effect of ϵ and path lengths on the ranking accuracy

We have computed the accuracy of the Reversed Local Path method (τ) under different ϵ value, as shown in Fig. S3. One can see that τ can achieve a maximum when ϵ is set to an optimal value. The optimal ϵ varies from one network to another and the setting of ϵ we used in the paper (i.e. $\epsilon = 0.1$) is not the optimal ϵ. However, this setting of ϵ can result in rather satisfactory ranking accuracy (i.e. $\epsilon = 0.1$ is near the optimal ϵ in many networks). In Fig. S3, we also marked the results when $\epsilon = \lambda = \langle k\rangle/(\langle k^2\rangle - \langle k\rangle)$ which is the infection probability we used for the SIR model. This setting of ϵ seems to be better than $\epsilon = 0.1$. As in real cases we usually don't know the true infection probability of the spreading process, we present the results of $\epsilon = 0.1$ in the paper.

In addition, we study the dependence of the accuracy (τ) on the length of paths (l) used in the RLP method, as shown in Fig. S4. One can see in the figure that at $l = 3$, the accuracy already reaches a plateau, which is why we only consider paths with length three in our method.

Supplementary Note 4: performance of RLP on networks with different sizes

We have examined the performance of our method in the modeled networks (i.e. SW [1] and BA [2] networks) with different sizes, as shown in Fig. S5. One can see that RLP can still significantly outperform LD when the network size is enlarged. Therefore, we believe that the advantage of the RLP method over the LD method will still be substantial in the network with very large size.

Supplementary Note 5: performance of RLP on networks with community structure

We study the effect of the community structure on our results. We use the well-known GN-benchmark network model [3]. The benchmark consists of 128 nodes, each with expected degree 16, which are divided into four groups of 32. A parameter k_{out} controls the average

number of links per node connecting to nodes outside its community. The larger $k_{\rm out}$ is, the less obvious the community structure is. The performance of different algorithms in this network is compared in Fig. S6 where we consider both random target scheme and local target scheme. In the random target scheme, 10% nodes (i.e. 12 nodes) are randomly selected as target nodes. In the local target scheme, 12 nodes within a community are randomly selected as target nodes. In Fig. S6, one can see that as $k_{\rm out}$ increases, the traditional centrality index (e.g. degree, betweenness, k-core) tends to have a better accuracy, while the RLP method's accuracy tends to decreases. In addition, we find that the RLP method generally performs better in the local scheme than the random scheme. These results indicate that the target spreading problem in general becomes more challenging and the advantage of RLP is bigger when the network diameter is large (e.g. the $k_{\rm out}$ is smaller in GN-benchmark).

Supplementary Note 6: spreading with local and global targets in BA networks

We compute ρ_i of each node in Barabasi-Albert (BA) networks [2] with size $N = 500$ and mean degree $\langle k \rangle = 4$. The dependence of ρ_i on the spreaders' degree in BA networks with the globalized target case and the localized target case is shown in Fig. S7(a)(b), respectively. In Fig. S7(a), i.e. the globalized target case, one can see that ρ_i strongly correlates with the spreaders' degree k_i. However, in the localized target case, the correlation between ρ and k is much weaker as shown in Fig. S7(b). For a fixed degree, there is a wide spread of ρ values, which indicates that degree is no longer a good predictor of nodes' spreading ability. In Fig. S7(b), the color of each point represents the mean shortest path length $\langle d_i \rangle$ from the spreader i to the target nodes. One can see that the nodes with small $\langle d_i \rangle$ and large k_i tend to have high ρ_i.

To further understand above observations, we investigate the effect of different location of the targets in Fig. S7(c)(d). We fix the number of target nodes as 30 and consider two scenarios, i.e. either the targets are randomly located in the network or they are located in a small area. To realize the second scenario, we first randomly pick up a node and set it as a center for this small area. The rest of the targets are placed in the nodes with the shortest path length not larger than 2 to the central node. We compare the fraction of infected target nodes ρ as a function of the infection probability λ in these two scenarios. As a benchmark, we also plot ρ versus λ with the globalized targets in both Fig. S7(c) and (d). One can see

that if the 30 targets are distributed randomly, the curve overlaps well with the curve of the globalized target case. However, when the targets are localized within two step distance, the ρ curve is a bit higher than two cases above. This is because when one tries to select the targets within $L = 2$ distance from a central target node, the large degree nodes are more likely to be selected. As they are easier to be infected in the spreading process, the fraction of infected nodes in this scheme is higher than the random/global scheme when the same infection probability λ is given. These results also indicate that the localization of the targets makes the spreading properties significantly differs from the traditional case.

Supplementary Note 7: the case when target nodes can be chosen as seeds

To get a more complete picture, we also consider some real cases where the target nodes can be chosen as seeds. The results are similar to those presented in this paper. The RLP method could also extend to this situation. Therefore, for the target nodes, the formula for RLP reads

$$S_{RLP} = \sum_{l=0}^{3} \epsilon^{l-1} f A^{l}, \tag{2}$$

where f is a $1 \times N$ vector in which the components corresponding to the target nodes are 1, and 0 otherwise. A is the $N \times N$ adjacency matrix of the network with $A_{ij} = 1$ indicating that node i connects to node j and $A_{ij} = 0$ otherwise.

In Fig. S8, we consider this situation and then compare the accuracy τ of the above-mentioned ranking methods under different infection probability λ. In this case, there are 30 randomly distributed targets in the network. Four networks are considered. In WS and BA networks (Fig. S8ab), we do not show the results of the k-core method as the k-shell values of all the nodes in these two networks are almost the same. The results in each figure is obtained by averaging over 5000 independent realizations. The procedure is that we first take a realization of a network, investigate lots of target node sets in order to compute τ, and then average τ over many network realizations. However, for each of the real network cases (Fig. S8cd), there is only one network and we just average the results over different target node sets. One immediate observation in Fig. S8 is that the RLP method has much higher accuracy τ than the other methods, especially when λ is small. However, when λ is too large and far exceeding the critical infection probability λ_c (marked by the orange vertical dashed lines in the figure), the spreading originated from each node may cover nearly the

whole network including the target nodes. In this case, the final spreading coverage can no longer reflect the true spreading ability of nodes. Therefore, the τ value of RLP is similar to that of the other three methods when λ is large. Compared with the Fig. 4 in the paper where the target node cannot be chosen as seeds, one can clearly see that the results are consistent, indicating the advantage of the RLP method over the existing methods.

Supplementary Note 8: the local degree method considering neighbors up to different distances l.

We investigate the spreading ability ranking accuracy τ under different m and L in four networks. At this point, LD_1 method represents that we only consider the degree of the nodes which are neighbors to the targets nodes, while LD_3 method includes the degree of nodes within the distance $l = 3$ from the target nodes (i.e. the LD method in text). We then compare the performance of the RLP method with this two kinds of LD methods in Fig. S9. The way we place the target nodes is the same as Fig. 2(b). We first select a node in the network as the so-called central node. There are m targets in the network and the $m - 1$ targets randomly locate in the nodes with maximum distance L (measured by the shortest path length) to the central node. Apparently, when L is infinitely large, these m nodes distribute randomly in the network. The smaller L is, the more localized the targets are. In Fig. S9, one can see that LD with $l = 3$ indeed outperforms LD with $l = 1$ in WS, Netsci and Y2H networks. However, LD with $l = 3$ has a lower accuracy than LD with $l = 1$ in BA networks. This is because the existence of the hub nodes in a BA network will make the target nodes neighbors up to a distance $l = 3$ cover almost all the nodes in the network. In this case, the LD with $l = 3$ method becomes similar to the traditional degree method and thus has a low accuracy.

We also notice that the accuracy of LD with $l = 3$ is rather stable under different m and L, which is different from that of LD with $l = 1$. For LD with $l = 1$, its accuracy tends to increases with m and L. This is because the number of target nodes neighbors increases with m and L. However, in LD with $l = 3$, the target nodes neighbors up to distance 3 have already cover most of the nodes in the local area. In this case, increasing m and L will not further increase the number of considered targets neighbors. Therefore, the accuary of LD with $l = 3$ is insensitive to the parameter m and L. However, both LD methods have lower

accuracy than our RLP method.

Supplementary Note 9: the relation between best spreaders and the highest rank in RLP method.

In order to study the relation between best spreaders and the values obtained from RLP method, we investigate two cases like Fig. 2(c)(d) in Netsci network. ρ represents the fraction of infected target nodes with the spreading originated from each node. V_{RLP} stands for each node's value calculated by the RLP method. One can clearly see that the Kendalls tau rank correlation coefficient is relatively high in Fig. S10, $\tau = 0.816$ in (a) and $\tau = 0.855$ in (b). Moreover, Fig. S10 also illustrates that the node with the highest value is also the best spreader.

Supplementary Figures

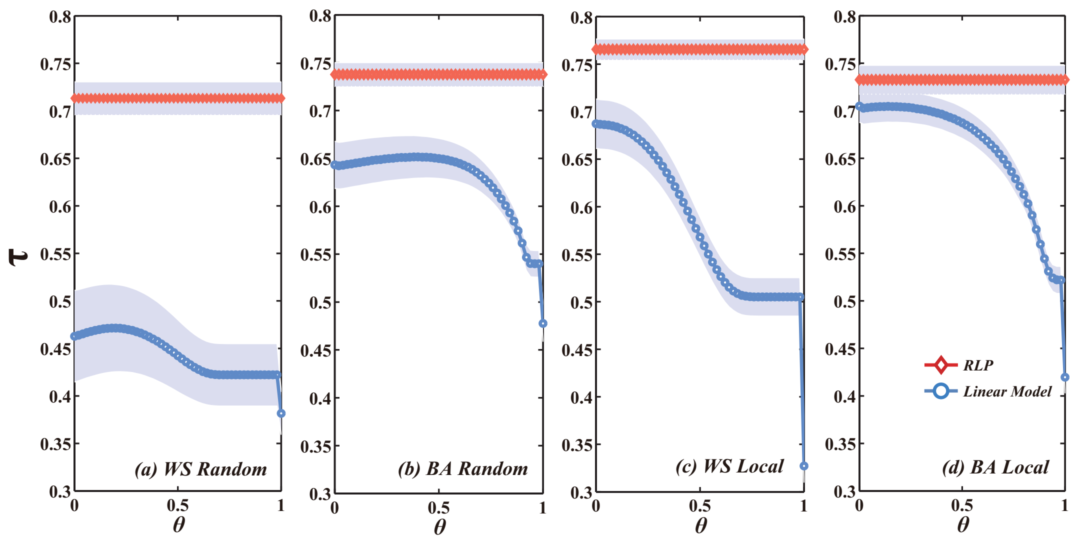

Supplementary Figure S 1: Kendall's tau rank correlation coefficient τ between the rankings obtained from a linear model and the true spreading ability ρ under different parameters θ. The red rhombus line represents the results of RLP method and the blue circle line represents the results of the linear model. Two networks are considered, i.e. (a)(c) WS, (b)(d) BA. In each network, we select 50 target nodes. In Fig. S1(a)(b), the targets are randomly located in the networks. In Fig. S1(c)(d), the targets are randomly locate within distance L=2 to a center node. In WS network, the infection probability λ=0.32, and in BA network, the infection probability λ=0.094. These two infection probabilities are all near the critical infection probability. The network parameters for BA and WS are $N = 500$ and $\langle k \rangle = 4$. The results in each figure is obtained by averaging over 5000 independent realizations.

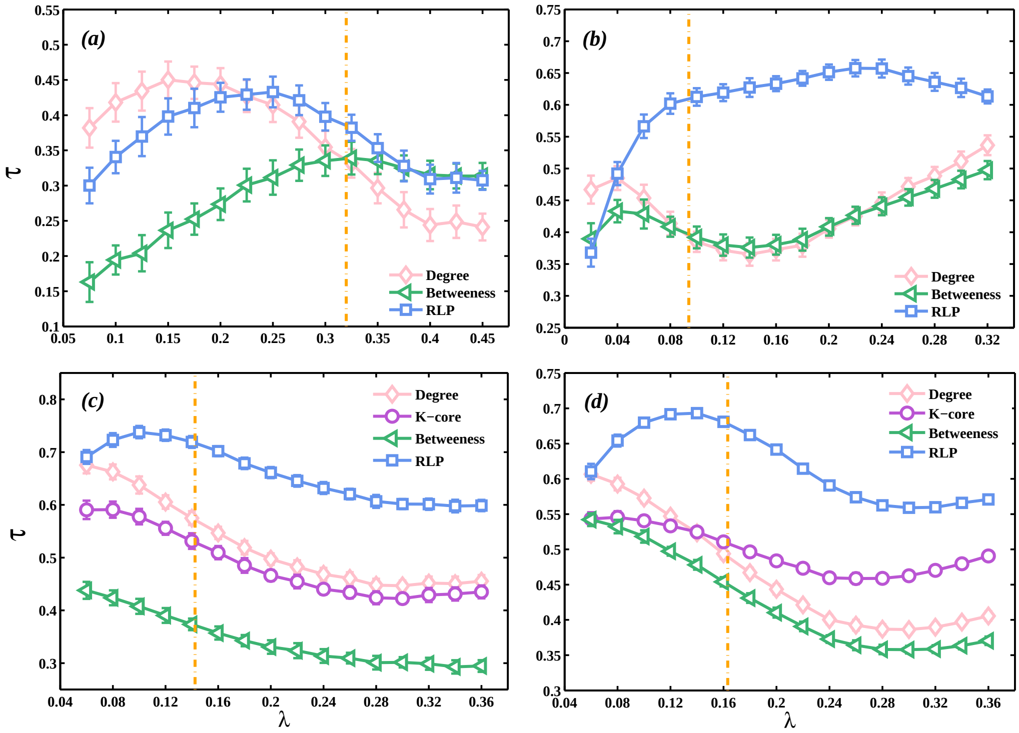

Supplementary Figure S 2: (Color online) Kendall's tau rank correlation coefficient τ between the rankings obtained from different methods and the true spreading ability ρ under different infection probabilities λ. Four networks are considered, i.e. (a) WS, (b) BA, (c) Netsci and (d) Y2H networks. In each network, all nodes are target nodes. Ranking methods include degree (pink diamonds), betweenness (green triangles), k-core (purple circles) or RLP (blue squares) methods. The orange dashed line corresponds to the critical infection probability. The results in each figure is obtained by averaging over 5000 independent realizations. In this figure, both WS and BA networks are with size N = 500 and mean degree $\langle k \rangle = 4$.

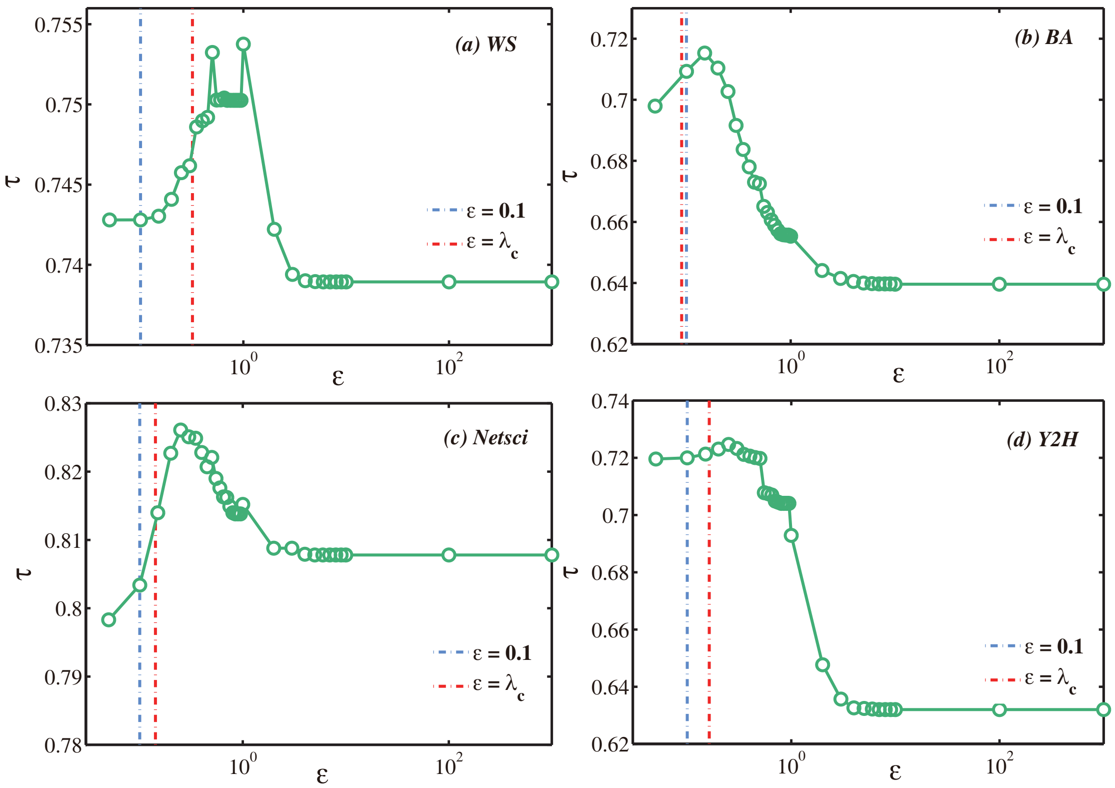

Supplementary Figure S 3: the accuracy (measured by τ) of the Reversed Local Path method under different ϵ value. In each network, 30 nodes are randomly selected as the target nodes. The results in each figure is obtained by averaging over 5000 independent realizations.

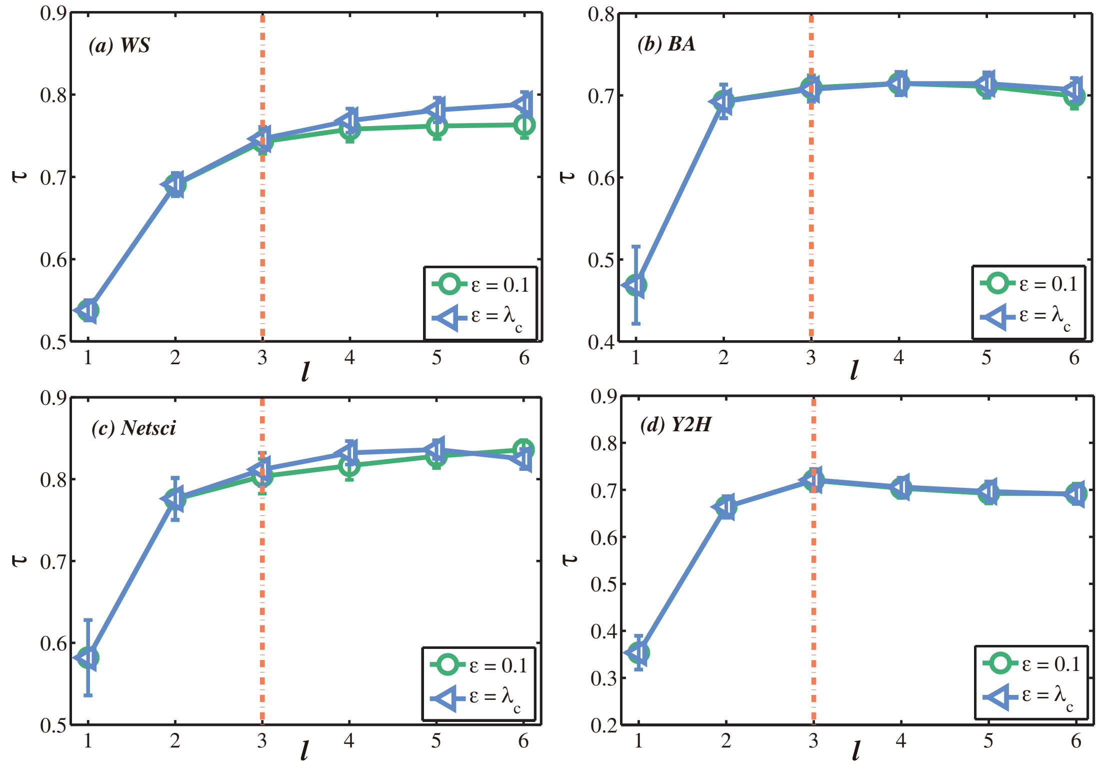

Supplementary Figure S 4: the dependence of the accuracy (τ) on the length of paths (l) used in the RLP method. In each network, 30 nodes are randomly selected as the target nodes. The results in each figure is obtained by averaging over 5000 independent realizations.

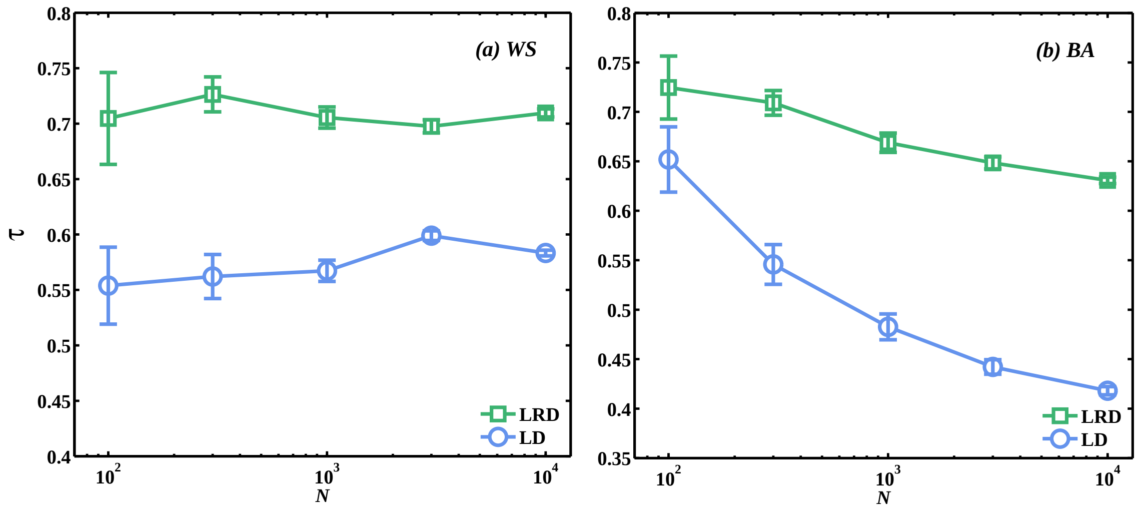

Supplementary Figure S 5: the accuracy (τ) of RLP and LD in the WS and BA networks with different sizes. In both WS and BA networks, the mean degree $\langle k \rangle = 4$. In each network, 10% nodes are randomly selected as the target nodes. The results in each figure is obtained by averaging over 5000 independent realizations.

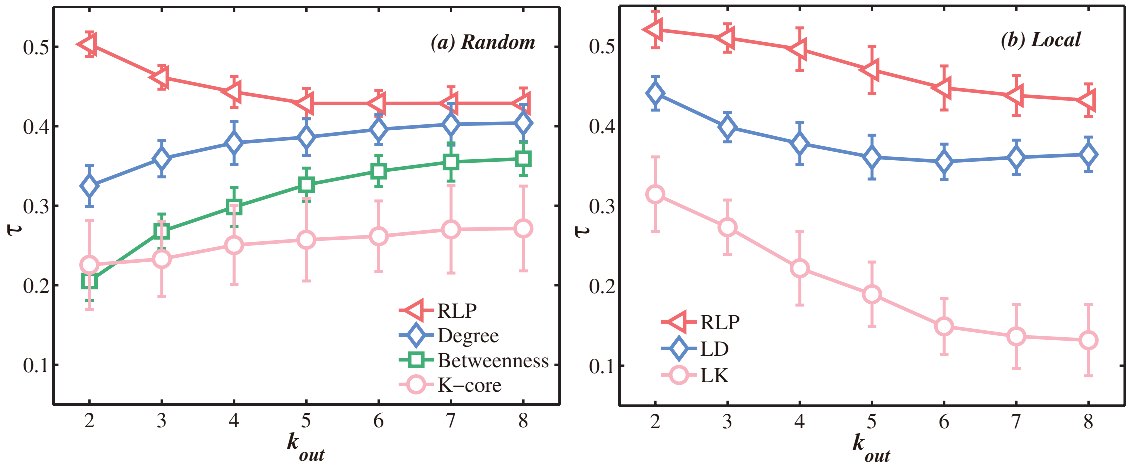

Supplementary Figure S 6: the accuracy of different methods in GN-benchmark networks with different k_{out}. In the random target scheme, 12 nodes (i.e. roughly 10% nodes) are randomly selected as target nodes. In the local target scheme, 12 nodes within a community are randomly selected as target nodes. The results in each figure is obtained by averaging over 5000 independent realizations.

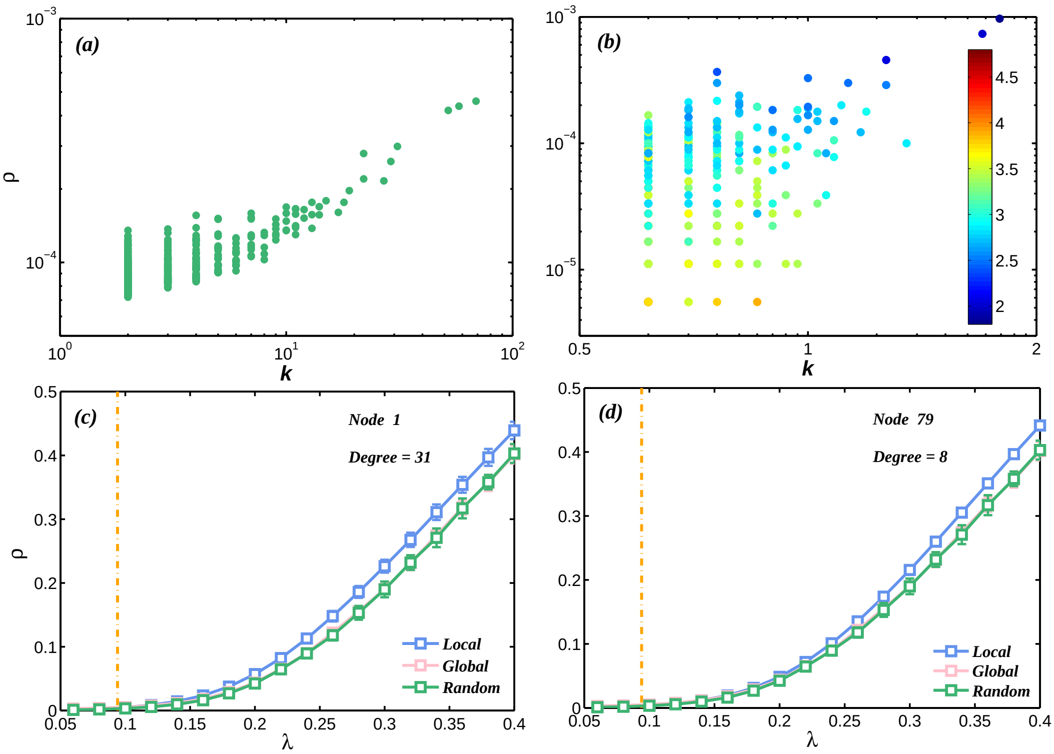

Supplementary Figure S 7: (Color online) (a) The dependence of the fraction of infected target nodes ρ on the initial spreaders' degree k. In this sub-figure, all the nodes in the network are target nodes. (b) The dependence of the fraction of infected target nodes ρ on the initial spreaders' degree k and the mean shortest path length $\langle d \rangle$ from the spreader to the target nodes. The color of each point represents the $\langle d \rangle$ of the spreader. In this sub-figure, there are only 30 target nodes. A node is randomly selected as a center and the rest of the targets are placed in the nodes with the shortest path length no larger than 2 to the center. In both (a)(b), the infection probability $\lambda = 0.06$, slightly smaller than the critical infection probability $\lambda_c = 0.094$. (c)(d) The fraction of infected target nodes ρ as a function of infection probability λ. In pink rhombus line, all the nodes in the network are target nodes. In green triangle line, we randomly select 30 nodes as the target nodes, while in blue square line, the target nodes are located the same as (b). The difference between (c) and (d) is that the center has $k = 31$ in (c) while $k = 8$ in (d). In all sub-figures, the networks are BA model with $N = 500$ and $\langle k \rangle = 4$. The results are obtained by averaging 500 independent realizations.

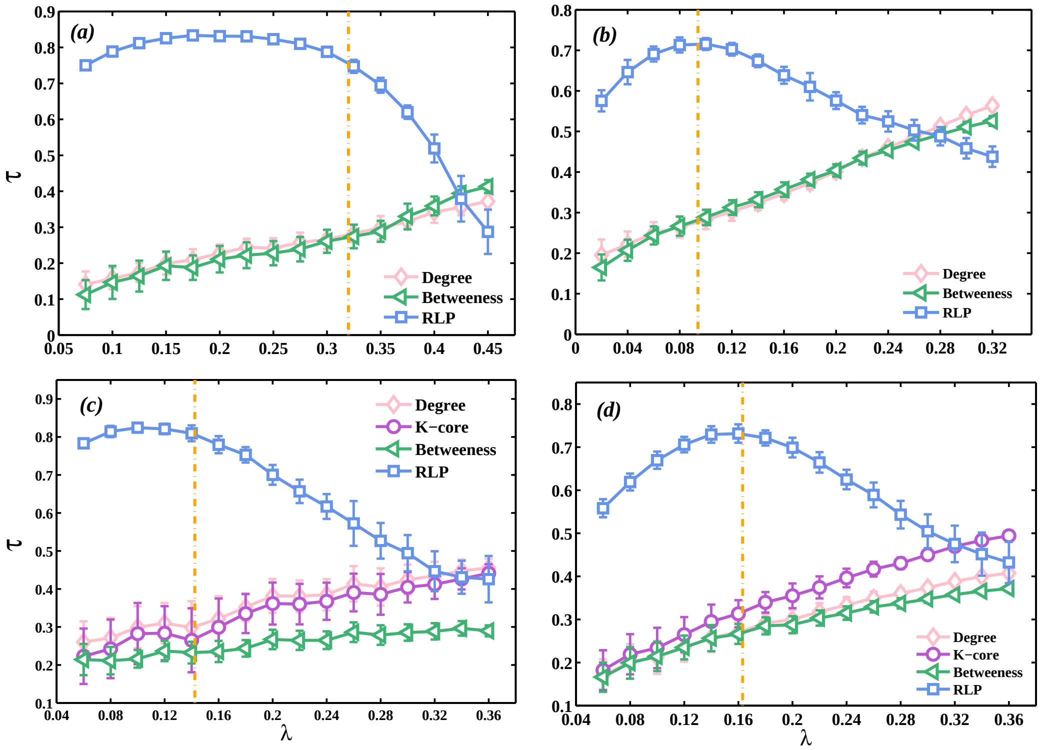

Supplementary Figure S 8: Kendall's tau rank correlation coefficient τ between the rankings obtained from different methods and the true spreading ability ρ under different infection probability λ. Four networks are considered, i.e. (a) WS, (b) BA, (c) Netsci and (d) Y2H networks. In each network, 30 target nodes randomly locate in the network. Ranking methods include degree (red diamonds), betweenness (green triangles), k-core (purple circles) or RLP (blue squares) methods. The orange dashed line corresponds to the critical infection probability. The results in each figure is obtained by averaging over 5000 independent realizations. In all sub-figures, the target nodes can also be chosen as seeds.

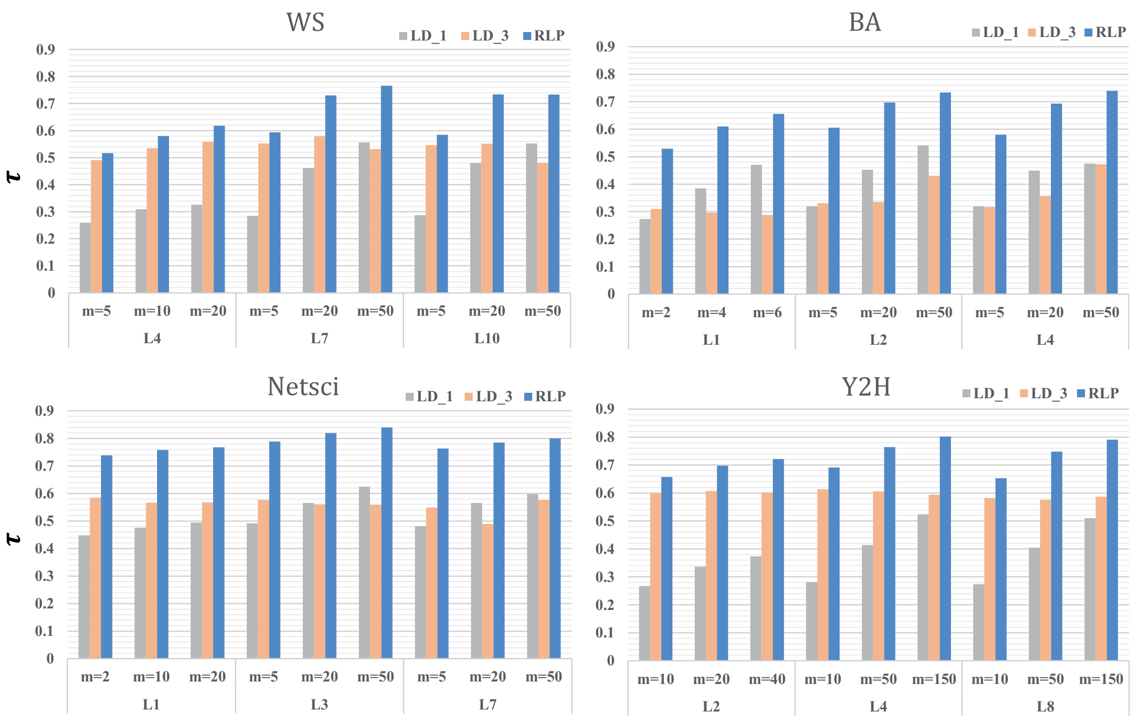

Supplementary Figure S 9: (Color online) The spreading ability ranking accuracy τ under different m and L in four networks. The parameters for WS and BA networks are $N = 500$ and $k = 4$. LD_1 method represents that we only consider the degree of the nodes which are neighbors to the targets nodes, while LD_3 method includes the degree of nodes within the distance l=3 from the target nodes. The results in this figure are obtained by averaging over 5000 independent realizations.

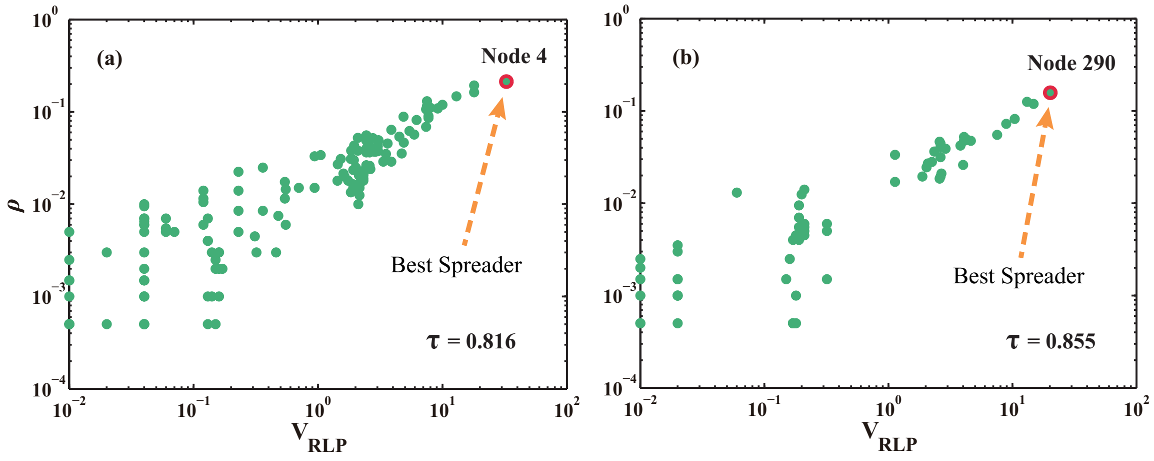

Supplementary Figure S 10: The relationship between the fraction of infected target nodes ρ and the values calculated by RLP method. In this figure, there are only 20 target nodes. A node is randomly selected as a center and the rest of the targets are placed in the nodes with the shortest path length no larger than 2 to the center. Center nodes are also target nodes. In both (a)(b), the infection probability $\lambda = 0.12$, slightly smaller than the critical infection probability $\lambda_c = 0.15$. The difference between (a) and (b) is that the center has $k = 27$ in (a) while $k = 8$ in (b). In these two sub-figures, the networks are Netsci with $N = 379$ and $\langle k \rangle = 4.8$.

[1] Watts D J and Strogatz S H 1998 Collective dynamics of 'small-world' networks. *Nature* **80**, 440-442

[2] Barabási A L and Albert R 1999 Emergence of scaling in random networks. *Science* **80**, 509-512

[3] Girvan M and Newman M E J 2002 Community structure in social and biological networks, PNAS **99**, 7821
